# Electrocatalytic CO_2_ reduction by a cobalt porphyrin mini-enzyme†

**DOI:** 10.1039/d4sc07026g

**Published:** 2025-02-25

**Authors:** Alison A. Salamatian, Jose L. Alvarez-Hernandez, Karishma B. Ramesh, Linda Leone, Angela Lombardi, Kara L. Bren

**Affiliations:** a Department of Chemistry, University of Rochester Rochester NY 14627-0216 USA kara.bren@rochester.edu; b Department of Chemical Sciences, University of Naples Federico II, Complesso Universitario Monte S. Angelo Via Cintia 80126 Naples Italy

## Abstract

Cobalt-mimochrome VI*a (CoMC6*a), a synthetic mini-enzyme with a cobalt porphyrin active site, is developed as a biomolecular catalyst for electrocatalytic CO_2_ reduction in water. The catalytic turnover number reaches ∼14 000 for CO production with a selectivity of 86 : 5 over H_2_ production under the same conditions. Varying the applied potential and the p*K*_a_ of the proton donor was used to gain insight into the basis for selectivity. The protected active site of CoMC6*a is proposed to enhance selectivity for CO_2_ reduction under conditions that typically favor H_2_ production by related catalysts. CoMC6*a activity and selectivity change only marginally under air, indicating excellent oxygen tolerance.

## Abstract 2

CoMC6*a (right) is a mini-enzyme with a cobalt porphyrin active site that favors CO_2_ over H^+^ reduction. Here, we provide evidence that its structure promotes selectivity compared to CoMP11-Ac (left), which has a solvent-exposed active site.

## Graphical abstract

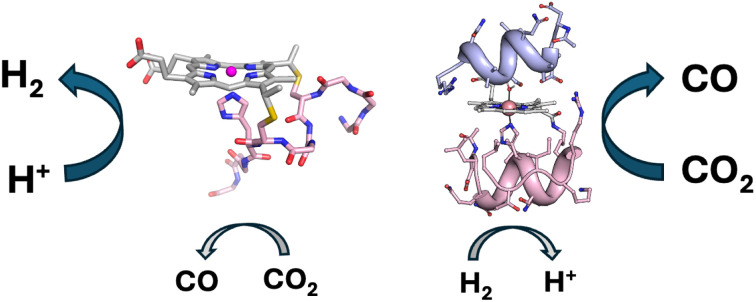

## Introduction

Electrochemical carbon dioxide (CO_2_) reduction is an appealing route to renewable fuel production.^1,2^ Achieving selectivity for CO_2_ reduction over proton reduction is an omnipresent challenge, since the reduction of CO_2_ to CO, or any stable product, requires protons (eqn (1) and (2)).^3,4^ Achieving selectivity in a protic solvent such as water is particularly challenging. However, there is significant interest in developing catalysis in water as an abundant source of protons and a desirable environmentally-friendly solvent.^5–7^ An additional challenge raised by use of water as a solvent is the poor solubility of CO_2_.^8,9^ Developing catalysts with microenvironments that sequester and activate CO_2_ in the presence of protons thus is of high interest.^10–15^

1 CO_2_ + 2H^+^ + 2e^−^ → CO + H_2_O

2 2H^+^ + 2e^−^ → H_2_

Nature's enzymes achieve high selectivity and activity for reactions such as CO_2_ reduction by providing an active-site microenvironment to promote substrate binding and transformation and by controlling electron and proton delivery.^16–19^ Inspired by Nature's catalysts, artificial enzymes for CO_2_ reduction (see examples in Table S1†) have been prepared by incorporation of synthetic CO_2_ reduction catalysts, such as [Ni(cyclam)]^2+^,^20^ Ni(terpyridine),^21^ or cobalt porphyrins,^22–24^ into proteins including azurin,^20^ cytochrome *b*_562_,^23^ myoglobin,^24^ an artificial protein αRep,^25^ or an engineered photosensitizer protein.^21^ Some of these systems have been reported to achieve enhanced activity^23^ and selectivity^20^ relative to the synthetic catalyst outside of the protein environment. For example, improved selectivity for CO_2_ over proton reduction by [Ni(cyclam)]^2+^ bound to the protein azurin was attributed to the protein scaffold providing restricting conformational flexibility of the catalyst and an active site buried within a solvent-excluded hydrophobic patch.^20^

Inspired by the importance of proton transfer steps in enzymatic catalysis,^17,26–29^ roles for endogenous^4,17,30–32^ and exogenous^7,22,30,33^ proton donors in determining CO_2_ reduction selectivity and activity have been proposed. The use of relatively weak Brønsted acids as proton donors is proposed to slow metal-hydride formation and thus disfavor the competing H_2_ evolution pathway.^1,34,35^ Electrochemical studies on an iron–porphyrin electrocatalyst^7^ and a cobalt macrocyclic catalyst^36^ showed that using a higher-p*K*_a_ buffer increases selectivity for CO over H_2_ production. Furthermore, in photochemical studies employing cobalt porphyrin catalysts, presence of a higher-p*K*_a_ buffer (bicarbonate, as opposed to phosphate) was shown to increase selectivity for CO over H_2_ production.^37,38^ Other properties of buffers have also been implicated in determining selectivity. For [Ni(cyclam)]^2+^, buffer steric properties and charges were found to impact selectivity for CO over H_2_ production; cationic buffers were proposed to stabilize an activated Ni–CO_2_ species in a second-sphere interaction, favoring CO production.^33^

**Fig. 1:**
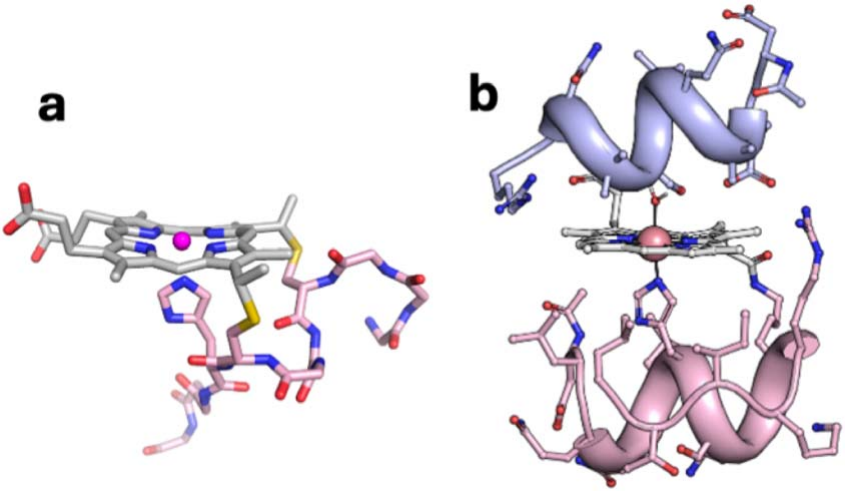
Models of (a) CoMP11-Ac; (b) CoMC6*a.

In a previous study, we reported CO_2_ reduction catalysis by a semisynthetic cobalt–porphyrin-containing mini-enzyme, CoMP11-Ac, consisting of a cobalt porphyrin with a covalently attached peptide donating an axial histidine ligand on the proximal side of the porphyrin (Fig. 1a). For CoMP11-Ac, selectivity for CO over H_2_ production in water is increased by using a higher-p*K*_a_ buffer as an exogenous proton donor, which is proposed to disfavor the formation of a metal-hydride species that yields H_2_. Furthermore, catalysis at a more negative potential (−1.4 V *vs.* Ag/AgCl/KCl_(1M)_) lowers selectivity for CO over H_2_ production, while applying a less negative potential (−1.2 V) increases selectivity.^22^

We now investigate effects of biocatalyst structure on selectivity for CO_2_*vs.* proton reduction. We have chosen a catalyst that, like CoMP11-Ac, has a cobalt porphyrin active site and axial His ligand, but that also has a peptide covering the distal side of the heme. This catalyst is a synthetic mini-enzyme, cobalt-mimochrome VI*a (CoMC6*a, Fig. 1b). Mimochromes are miniaturized porphyrin-based metalloproteins consisting of a deuteroporphyrin sandwiched between two peptide chains covalently bound to the porphyrin.^39,40^ MC6*a is a proven framework for catalysis, displaying peroxidase,^41–43^ peroxygenase^42–44^ or hydrogenase^45,46^ activities depending on conditions and the metal ion. Its scaffold consists of a distal decapeptide and a proximal tetradecapeptide that provides the axial His ligand to the metal ion. Helical secondary structure is favored by the inclusion of two 2-aminoisobutyric acid residues in the distal peptide.^47^

Previously, CoMC6*a was shown to act as an electrocatalyst for H_2_ evolution from water with a turnover number (TON) exceeding 230 000 (ref. 45) as well as a catalyst in a system for photochemical H_2_ evolution.^46^ Subsequent studies of CoMC6*a catalysis of H_2_ evolution from water revealed that buffer acid species play a critical role in proton delivery to CoMC6*a during catalysis, with their structures and p*K*_a_ values impacting catalytic rate, potential, and mechanism.^48^ In particular, proton-coupled electron transfer (PCET) was shown to be required for H_2_ production by CoMC6*a, with the catalytic potential shifting with the p*K*_a_ of the buffer acid in a Nernstian fashion. Furthermore, catalytic rate was shown to depend on buffer sterics, an observation attributed to the impact of the distal peptide in hindering proton delivery by protonated buffer.^48^ Interestingly, the specific effects of buffer acid on H_2_ production catalysis differ from those observed for CoMP11-Ac, for which buffer p*K*_a_, but not buffer structure, plays a role in determining catalytic rate, likely as a result of the solvent-exposed active site of CoMP11-Ac.^49^

Having observed these impacts of catalyst structure on H_2_ evolution catalysis by CoMP11-Ac *vs.* CoMC6*a, we now turn to investigating the impact of structure on CO_2_ reduction by CoMC6*a. We hypothesized that the more hydrophobic and enclosed active site of CoMC6*a would favor CO_2_ reduction. Using conditions applied to CoMP11-Ac to facilitate comparison, the roles of both applied potential and exogenous proton donor p*K*_a_ in determining CO_2_*vs.* proton reduction selectivity and activity by CoMC6*a are investigated. Comparison to previous results on CoMP11-Ac indicates that the distal peptide plays a role in enhancing selectivity for CO_2_ reduction. Finally, we demonstrate that this catalyst exhibits excellent tolerance for oxygen, with minimal impact on CO_2_ reduction activity or selectivity.

## Results and discussion

CoMC6*a was prepared and characterized as described in the ESI (Fig. S1 and S2)† as well as previous publications.^45,47^ Cyclic voltammetry (CV) of 1 μM CoMC6*a was carried out using a hanging mercury drop electrode, used in previous related work.^22,45,48^ As was observed for CoMP11-Ac,^22^ dip-and-stir experiments^50^ indicate that CoMC6*a adsorbs to the electrode, acting as an immobilized catalyst (Fig. S3 and S4†).

### Effects of applied potential

**Fig. 2:**
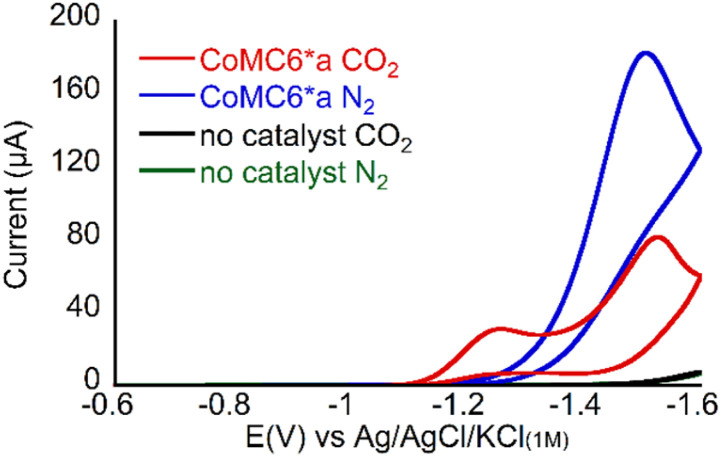
Cyclic voltammograms of 1 μM CoMC6*a pH 5.9 in 50 mM MOPS, 0.1 M KCl, at 100 mV s^−1^, scan 2, under 1 atm of the indicated gas.

CV of 1 μM CoMC6*a at pH 6 in 50 mM 3-morpholinopropane-1-sulfonic acid (MOPS, p*K*_a_ 7.2) under N_2_ (Fig. 2) shows faradaic current beginning at an onset potential of ∼ −1.2 V *vs.* Ag/AgCl/KCl_(1M)_ (all potentials reported herein are reported against this reference). The rise in current forming a single peak is attributed to CoMC6*a electrocatalytic H_2_ evolution activity *via* protonated buffer consumption, which was previously reported under similar conditions.^45,48^ When the solution is saturated with CO_2_ and placed under 1 atm CO_2_, two peaks are observed at ∼ −1.2 V and ∼ −1.5 V (Fig. 2). The resulting increase in current at ∼ −1.2 V may indicate selective CO_2_ reduction over proton reduction at this potential. Furthermore, the anodic shift of the catalytic onset potential may be due to CO_2_ coordination and reduction or a coupled EC/CE reaction.

To characterize product formation, controlled potential electrolysis (CPE) experiments were run on 1 μM CoMC6*a in the presence of MOPS for two hours, after which the headspace gas was sampled and analyzed by gas chromatography (GC). Experiments were run at −1.2 and −1.4 V to aid comparison to published results on CoMP11-Ac at these conditions (Table S2†).^22^ At −1.4 V under N_2_ with no CO_2_ present, H_2_ is produced with nearly quantitative faradaic efficiency (FE_H_2__ 96 ± 4%), consistent with previous results.^45,48^ When a CO_2_-saturated solution of CoMC6*a under one atmosphere of CO_2_ is subjected to CPE, the major product is CO (Tables 1, S3 and Fig. S5†). However, selectivity for CO formation over H_2_ under these conditions changes with applied potential, with higher selectivity (85 : 6 FE_CO_ : FE_H_2__) at −1.2 V compared to 68 : 24 at −1.4 V (Tables 1, S3 and Fig. S5†). The turnover number (TON) for CO production also is dependent on potential, with double the value (2200 ± 300) at the less cathodic potential of −1.2 V. In comparison with results on CoMP11-Ac under the same conditions (Table S2†), FE_CO_ (85 ± 2%) and FE_H_2__ (8 ± 2%) are nearly the same as the values for CoMC6*a at −1.2 V. However, at −1.4 V (Table 2), CoMP11-Ac favors H_2_ production, with FE_CO_ of 21 ± 5% and FE_H_2__ of 63 ± 13%. Thus, under these conditions at −1.4 V, CoMC6*a shows significantly greater selectivity for CO_2_ over proton reduction compared to CoMP11-Ac, supporting the hypothesis that protection of the CoMC6*a active site by the distal peptide enhances selectivity.

**Table 1:** Results of CPE experiments on CoMC6*a^a^

Gas	Buffer	*E* b (V)	FE_(H_2_)_ %	FE_(CO)_ %	TON_(H_2_)_	TON_(CO)_	*Q*_T_ (C)
CO_2_	CAPS (p*K*_a_ 10.4)	−1.4	4 ± 1	76 ± 10	110 ± 20	2100 ± 600	2.6 ± 0.4
		−1.2	4 ± 4	73 ± 5	11 ± 10	230 ± 10	0.3 ± 0.1
CO_2_	CHES (p*K*_a_ 9.3)	−1.4	14 ± 1	67 ± 12	280 ± 10	1300 ± 400	1.9 ± 0.1
		−1.2	11 ± 1	86 ± 11	100 ± 20	800 ± 200	0.9 ± 0.1
CO_2_	MOPS (p*K*_a_ 7.2)	−1.4	24 ± 4	68 ± 8	390 ± 120	1100 ± 200	1.6 ± 0.5
		−1.2	6 ± 1	85 ± 11	160 ± 40	2200 ± 300	2.5 ± 0.2
N_2_	CAPS (p*K*_a_ 10.4)	−1.4	88 ± 10	∼0	1100 ± 400	∼0	1.2 ± 0.3
		−1.2	No above-background activity^c^				
N_2_	CHES (p*K*_a_ 9.3)	−1.4	97 ± 14	∼0	1800 ± 200	∼0	1.8 ± 0.1
		−1.2	78 ± 14	∼0	130 ± 30	∼0	0.2 ± 0.1
N_2_	MOPS (p*K*_a_ 7.2)	−1.4	96 ± 4	1.0 ± 0.3	3900 ± 1500	45 ± 12	3.9 ± 1.4
		−1.2	No above–background activity				

a Two-hour CPE experiments conducted on 1 μM catalyst in 0.5 M buffer with 1 M KCl. Data shown corresponds to the average of at least three individual runs, the error corresponds to the difference between the average and the replicate with the greatest difference from the average; ESI shows detailed results. The pH of all MOPS, CHES, and CAPS solutions after purging with CO_2_ was 6.5 ± 0.2; and 7.2 ± 0.2 when purged with N_2_.

b Potentials reported *vs.* Ag/AgCl/KCl_(1M)_.

c Activity is not reported if it did not exceed three times background in more than one replicate.

**Table 2:** FE values for CoMP11-Ac and CoMC6*a at −1.4 V^a^

Buffer	Catalyst	FE_(H_2_)_ %	FE_(CO)_ %
CAPS (p*K*_a_ 10.4)	CoMP11-Ac	29 ± 6	48 ± 10
	CoMC6*a	4 ± 1	76 ± 10
CHES (p*K*_a_ 9.3)	CoMP11-Ac	43 ± 9	57 ± 4
	CoMC6*a	14 ± 1	67 ± 12
MOPS (p*K*_a_ 7.2)	CoMP11-Ac	63 ± 13	21 ± 5
	CoMC6*a	24 ± 4	68 ± 8

a Data on CoMP-11 from ref. 22 Data collected under 1 atm CO_2_, 0.5 M buffer, pH 6.5. Full table of comparative results in ESI.

### Effects of proton donor p*K*_a_

**Fig. 3:**
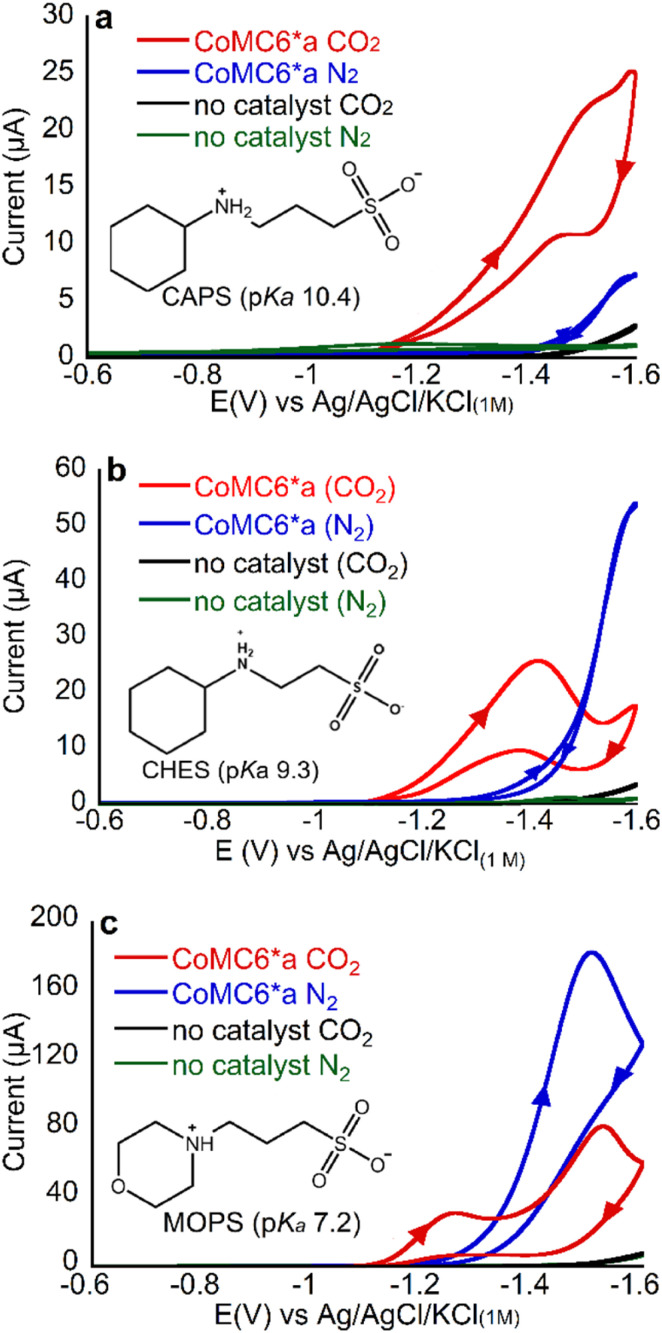
CVs of 1 μM CoMC6*a in 50 mM (a) CAPS, (b) CHES, (c) MOPS. For all CVs, pH = 5.9, [KCl] = 0.1 M and scan rate = 100 mV s^−1^. Arrows in the CV traces indicate the scanning direction.

An important tool for addressing product selectivity and gaining mechanistic insights in CO_2_ reduction electrocatalysis is tuning proton donor properties.^31,51^ For a number of catalysts in water, protonated buffers have been shown to be the primary proton donors in proton-requiring catalysis (except at low pH values)^52^ for H_2_ production^48–50^ and CO_2_ reduction,^7,33,49^ with buffer properties impacting catalytic rate, mechanism, and selectivity.^36–38^ For CoMC6*a, properties of buffer acids have been shown to impact electrocatalytic H_2_ evolution efficiency, activity, and mechanism: lower-p*K*_a_ buffers result in an anodic shift in the catalytic wave, which has been attributed to their role in PCET,^48^ and less bulky buffers increase catalytic current, a phenomenon attributed to distal CoMC6*a peptide hindering proton donor access to the active site.^48^ To determine the effect of proton donor on CO_2_ reduction selectivity by CoMC6*a, we chose three structurally related buffers: MOPS, used above (p*K*_a_ = 7.2), *N*-cyclohexyl-2-aminoethanesulfonic acid (CHES, p*K*_a_ = 9.3) and 3-(cyclohexylamino)-1-ethanesulfonic acid (CAPS p*K*_a_ = 10.4; structures are shown in Fig. 3).

First, we collected CVs of CoMC6*a under N_2_ or CO_2_, with the solution saturated with the respective gas. Under N_2_, there is only one feature, which is between −1.4 and −1.6 V, and was previously shown to be associated with catalytic H_2_ evolution.^45,48^ The peak current of this low-potential feature decreases with increasing buffer p*K*_a_, consistent with lower H_2_ production activity with less acidic proton donors (Fig. 3).

Under CO_2_, the CV changes dramatically but in a manner dependent on the buffer present. With all three buffers, an increase in current under CO_2_ relative to that under N_2_ is seen at ∼ −1.2 V, a potential at which CPE experiments show (*vide infra*) there is minimal H_2_ production (Fig. 3 and Table 1). This result suggests that there may be enhanced CO_2_ reduction ∼ −1.2 V.

To determine products formed, two-hour CPE experiments on CoMC6*a in MOPS, CHES and CAPS buffers at pH 6 were performed at −1.2 and −1.4 V, with results in Tables 1, S3–S5 and Fig. S5–S7.† The UV-vis spectrum of the catalyst in bulk solution shows minimal change before and after CPE, indicating catalyst robustness (Fig. S8†). Under N_2_ at −1.2 V, no activity above background was observed in the presence of CAPS or MOPS, and very low activity was observed in CHES, indicating that minimal H_2_ production occurs at −1.2 V in the presence of all three buffer acids under these conditions, consistent with prior results on CoMC6*a.^48^ At −1.4 V under N_2_, the charge passed exceeds background for all three buffers, with H_2_ formation with FE_H_2__ values from 88 to 97%. As we lower buffer p*K*_a_, we see an increase in TON_H_2__, supporting the hypothesis that more acidic proton donors enhance H_2_ production activity, in line with prior results.^48^

When CPE of CoMC6*a is performed under CO_2_, CO becomes the major product under all conditions used here. At −1.2 V under CO_2_, FE_CO_ is approximately the same for experiments run with the three different buffer acids (ranging from 73 to 85%) and the FE_H_2__ values are also similar (4–11%), indicating that the p*K*_a_ of the buffer does not have a significant impact on selectivity at −1.2 V. In contrast, at −1.4 V under CO_2_, FE_H_2__ increases from 4 ± 1% to 14 ± 1% to 24 ± 4% as buffer p*K*_a_ decreases, showing that increased buffer acidity enhances H_2_ evolution under a CO_2_ atmosphere, possibly by promoting formation of a metal hydride or its protonation. FE_CO_ shows minimal change with buffer p*K*_a_ at −1.4 V, (67–76%), indicating that the effect of increased buffer p*K*_a_ on enhancing selectivity for CO production at −1.4 V results primarily from decreasing H_2_ production.

Comparison to results on CoMP11-Ac (Fig. 1) provides insight into how catalyst structure impacts selectivity. Similar to CoMC6*a, at −1.2 V, CO : H_2_ selectivity of CoMP11-Ac shows no dependence on buffer acid p*K*_a_ (Table S2†). At −1.4 V, also like CoMC6*a, CoMP11-Ac shows an increase in selectivity for CO_2_ reduction over proton reduction as the p*K*_a_ of the buffer acid is increased (Table 2 and S2†).^22^ CoMP11-Ac and CoMC6*a thus show similar trends in CO : H_2_ selectivity with buffer acid p*K*_a_, with no dependence at −1.2 V and an increased FE_CO_ : FE_H_2__ with decreased buffer acidity at −1.4 V, dominated by an impact on FE_H_2__. However, CoMC6*a has a higher CO : H_2_ selectivity under all conditions, always in favor of CO_2_ reduction. These results indicate that the CoMC6*a structure enhances CO_2_ reduction selectivity over proton reduction, an effect primarily seen at the more negative potential used herein.

For CoMP11-Ac, two mechanisms were proposed at the two different potentials.^22^ At −1.4 V, a mechanism invoking formal Co(i) formation was proposed, consistent with an estimated Co(ii/i) reduction potential of −1.42 V.^52^ Cobalt hydride is proposed to yield H_2_ upon protonation, and this process accounts for the greater FE_H_2__ at a more negative potential. This mechanism is in line with the observed selectivity dependency on the buffer acid p*K*_a_ at −1.4 V, as a more acidic proton donor will favor Co(i) protonation,^48^ thus biasing the system toward H_2_ formation. At −1.2 V, a mechanism in which CO_2_ binding couples to electron transfer to form a formal Co(i)–CO_2_ adduct was invoked, which avoids directly forming a Co(i) species and accounts for the lack of dependence of selectivity on buffer p*K*_a_ at this potential. This mechanism has a selectivity-determining step prior to any protonation step, which suggests that selectivity will not depend on proton donor p*K*_a_, in line with the experimental results at −1.2 V.

**Fig. 4:**
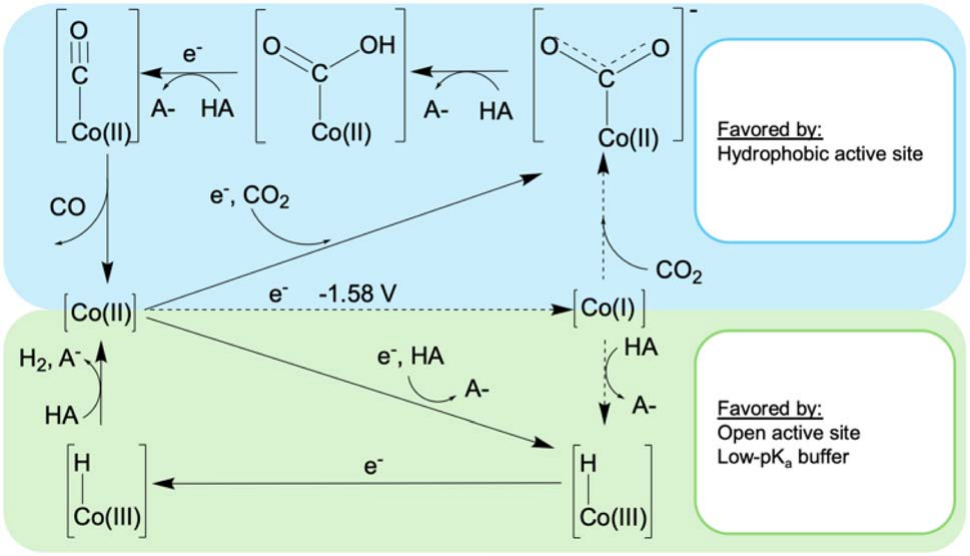
Proposed mechanisms for H_2_ and CO formation catalyzed by CoMC6*a. The dotted lines indicate processes not observed or expected under the conditions used herein.

To consider this model for CoMC6*a, we measured the formal Co(ii/i) reduction potential. This was accomplished under N_2_ at high pH and with a rapid scan rate, conditions at which H_2_ evolution is suppressed. From quasi-reversible CVs at pH 10–12, a midpoint potential of ∼ −1.58 V was measured (Fig. S9†). Thus, under the conditions used here for catalysis, direct formation of Co(i) is not possible. For CO_2_ reduction, reaching this formal oxidation state will require CO_2_ binding before or coupled with reduction. For proton reduction, PCET is required, as was previously demonstrated.^48^ These observations lead to the proposed mechanism in Fig. 4, which has its basis in published mechanisms for CO_2_ reduction and proton reduction by cobalt porphyrins.^53^ However, the low potential of Co(ii/i)MC6*a precludes direct formation of a Co(i) species under these conditions, a process typically invoked in related systems.^22,37,53^ To provide additional data to test this model, effects of CO_2_ concentration on catalysis were measured.

### Effects of CO_2_ partial pressure

**Fig. 5:**
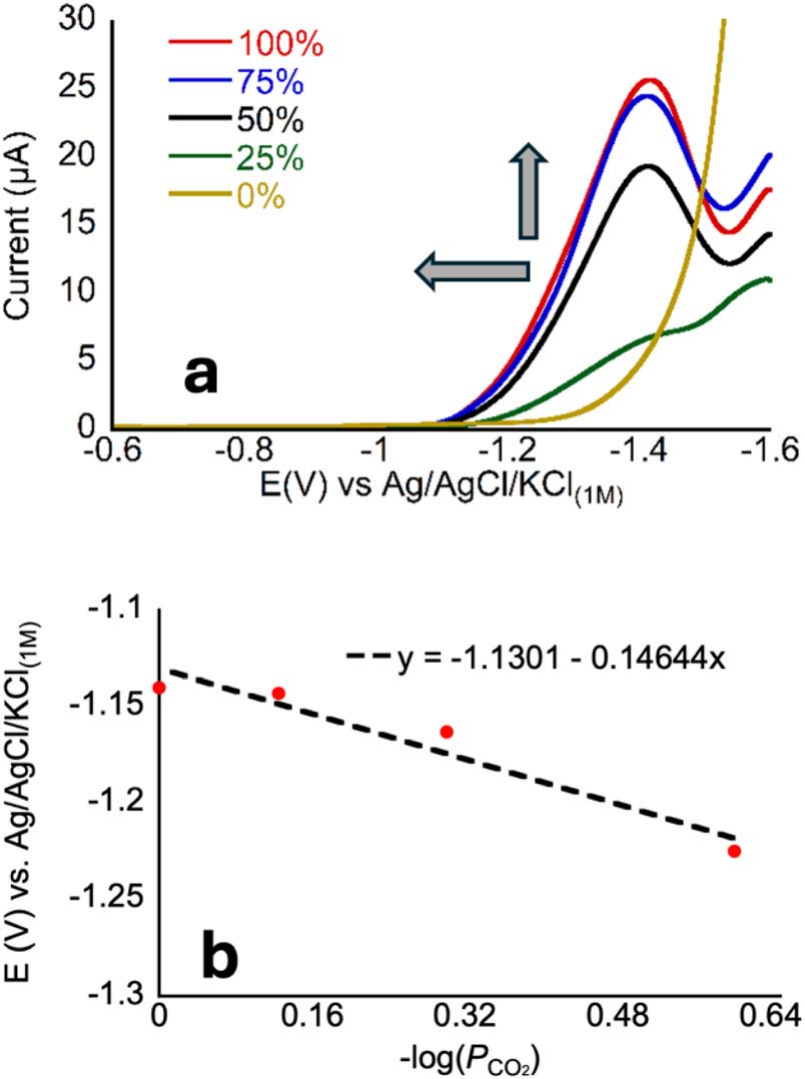
(a) Linear sweep voltammograms of 1 μM CoMC6*a in 50 mM CHES, 0.1 M KCl, pH 5.9 ± 0.1 at 100 mV s^−1^ under different *P*_CO_2__, the arrows indicate the direction of increasing *P*_CO_2__. (b) Plot of *E*_i_*vs.* −log(*P*_CO_2__) showing a slope of ∼150 mV per decade. *R*^2^ = 0.94.

Prior experiments examined the effect of proton donor (buffer) concentration on catalysis. Next, we examined effects of CO_2_ by collecting voltammograms as a function of CO_2_ partial pressure (*P*_CO_2__).^22^ In the presence of increasing partial pressures of CO_2_ (Fig. 5), a CV wave develops on the anodic side of the voltammogram, consistent with a process that is dependent on the concentration of CO_2_. The proposed mechanism, invoking coupled CO_2_ binding and reduction, should be dependent on the following equation under equilibrium conditions. Note that *E*_h_ refers to the half-wave potential:

3 M + e^−^ + CO_2_ ⇄ [M–CO_2_]^−^

4
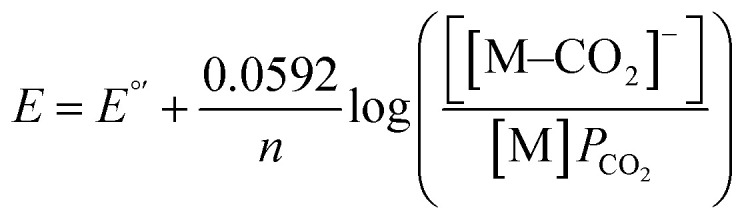

5 *E*_h_ = *E*°′− 0.0592 log(*P*_CO_2__)

To analyze these data, we chose a current near the foot of the wave (1.5 μA) to reflect the CO_2_-dependent process that occurs at less cathodic potentials than H_2_ production because a distinct peak is not always present in the voltammograms of CoMC6*a. We then define *E*_i_ as the potential at which this current is reached; we have used this approach when *E*_h_ (eqn (5)) cannot be readily defined (Fig. 5).^22^

6 *E*_i_ = −0.0592 log(*P*_CO_2__) + *E*°′

The negative non-zero slope seen in Fig. 5 reflects the increasing current with increasing *P*_CO_2__, consistent with a relationship between CO_2_ concentration and electron transfer, which supports our proposed mechanism. However, because a clear peak is not present reflecting primarily CO_2_ reduction, defining a quantitative relationship is not possible from these data.

Examination of Fig. 5a reveals that the voltammogram is nearly the same under 75% and 100% CO_2_, which contrasts with the clear changes from 0 to 75%. This change in dependence suggests that, above 75%, substrate (CO_2_) availability is no longer a limiting factor in catalysis. Notably, this observation differs from what is seen for CoMP11-Ac, for which the anodic shift continues for all *P*_CO_2__ values in the same range. To determine whether the proton donor becomes limiting under these conditions, we measured CVs for CoMC6*a under a CO_2_ atmosphere under varied concentrations of CHES buffer (the buffer used in Fig. 5). In contrast with the increase in catalytic current seen as a function of [CHES] (and all buffers)^48^ under N_2_, the CVs under CO_2_ are nearly invariant as a function of [CHES] (Fig. S10 and S11†). These observations for CoMC6*a indicate that, in the presence of CO_2_, a process other than CO_2_ or proton delivery limits catalysis. This may be a conformational rearrangement of the catalyst, *i.e.*, of the distal peptide to facilitate substrate access, or a later step in catalysis such as C–O bond breakage.

### Effect of air on catalysis

**Fig. 6:**
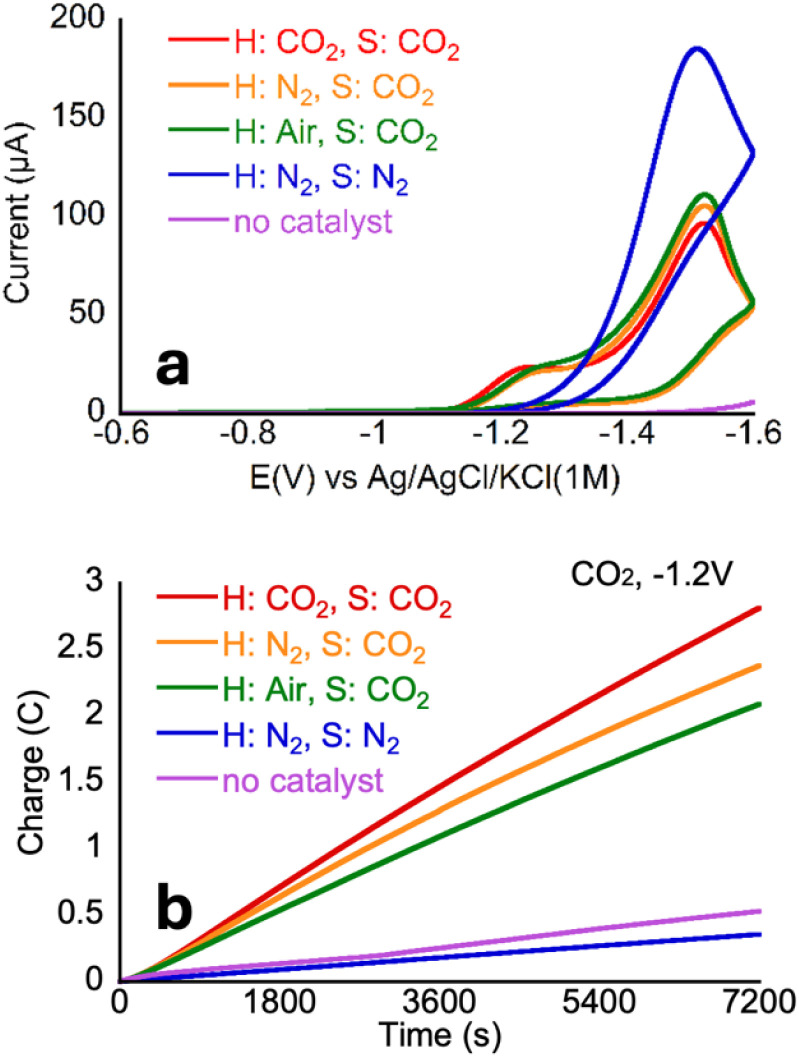
(a) CVs of 1 μM CoMC6*a in 50 mM MOPS, pH 5.9 ± 0.1. For all CVs, [KCl] = 0.1 M and scan rate = 100 mV s^−1^. Arrows in the CV traces indicate the scanning direction. (b) CPE experiments run in 0.5 M MOPS, 1 M KCl, the concentration of catalyst was 1 μM when present. The pH of all MOPS after purging with CO_2_ was 6.5 ± 0.1; and 7.2 ± 0.2 when purged with N_2_. Potentials reported *vs.* Ag/AgCl/KCl_(1M)_. H: headspace S: solution.

Since practical sources of CO_2_ such as flue gas tend to have impurities such as oxygen (O_2_), which has been shown to negatively affect many CO_2_ reduction catalysts, developing catalysts that can facilitate CO_2_ reduction in the presence of oxygen is a priority.^54^ To test whether O_2_ impacts CO_2_ reduction catalysis by CoMC6*a, a CV of a CoMC6*a solution saturated with CO_2_ was collected under room air (Fig. 6). The CV of CoMC6*a was not significantly impacted by the presence of air, overlaying closely with CVs under CO_2_ and nitrogen, suggesting the possibility of air-tolerant CO_2_ reduction. Results were similar for CVs of CoMC6*a solutions saturated with CO_2_ whether under 1 atmosphere of CO_2_, N_2_, or air. Next, two-hour CPEs were run to determine the impact of air on product formation. The resulting CPEs (Fig. 6 and Table 3) showed no significant difference in selectivity. The overall charge passed and TON values decreased when CO_2_ was removed from the headspace. This observation is consistent with lower activity with a decrease in available substrate and demonstrates an effect of changing the headspace on the two-hour CPE experiment. These results indicate that CoMC6*a maintains CO_2_ reduction activity and selectivity in the presence of O_2_. Note that air tolerance for H_2_ evolution by CoMC6*a was previously demonstrated.^45^

**Table 3:** Results of CPE experiments on CoMC6*a in the presence and absence of air^a^

GAS_Headspace_	GAS_Solution_	*E* b (V)	FE_(H_2_)_ %	FE_(CO)_ %	TON_(H_2_)_	TON_(CO)_	*Q*_T_ (C)
CO_2_	CO_2_	−1.2	6 ± 1	85 ± 11	160 ± 40	2200 ± 300	2.5 ± 0.2
Air	CO_2_	−1.2	4 ± 1	86 ± 7	67 ± 30	1500 ± 500	1.7 ± 0.6
N_2_	CO_2_	−1.2	5 ± 4	90 ± 10	80 ± 60	1500 ± 200	1.6 ± 0.1
N_2_	N_2_	−1.2	No above-background activity^c^				

a Two-hour CPE experiments conducted on 1 μM catalyst in 0.5 M MOPS with 1 M KCl. Results correspond to the average of at least three individual runs, the error corresponds to the difference between the average and the replicate with the greatest difference from the average. The pH of all solutions was adjusted to 6 for experiments. CPEs under air were purged with CO_2_ before the headspace was replaced with air ∼99% of the CO_2_ was replaced.

b Potentials reported *vs.* Ag/AgCl/KCl_(1M)_.

c Activity is not reported if it did not exceed three times background in more than one replicate.

While more investigations are needed to understand the basis for this air tolerance, there are a few reported examples that provide context. One is a cobalt phthalocyanine catalyst anchored to carbon nanotubes for CO_2_ reduction. In this system, FE_CO_ drops from 93% to 0% in the presence of 5% O_2_. However, protecting the cobalt phthalocyanine with a bioinspired polymer of intrinsic microporosity increased FE_CO_ in the presence of 5% O_2_ to 75.9%. At levels of O_2_ in air of 22%, however, FE_CO_ decreased to 49.7%.^55^ Another oxygen-tolerant transition-metal catalyst for CO_2_ reduction is an iron–porphyrin catalyst with four ferrocenes in its distal site that displays a 500-fold faster rate of CO_2_ binding compared to O_2_ binding, giving the catalyst high FE_co_ of 84% in the presence of 25% O_2_.^56^ Its O_2_ tolerance is also attributed to its favorable 4-electron reduction of O_2_ to H_2_O that avoids the formation of destructive reactive oxygen species, as well as rapid CO_2_ binding.^56^

### Insights into effects of catalyst structure on activity

Nature's enzymes have enviable properties, typically rapid catalysis, high substrate and product specificity, and great efficiency (*i.e.* low overpotential). These properties are attributed to the active-site microenvironment provided by the polypeptide matrix.^16,57^ However, Nature's metalloenzymes can be challenging to isolate in significant quantities and often are large structures with a low density of active sites. Furthermore, many enzymes that make H_2_ and that reduce CO_2_ are sensitive to oxygen. Thus, there has been interest in developing biomolecular catalysts that are relatively easy to prepare and work with, but retain the advantage of having polypeptide matrix that can be tuned to engineer the active site environment.^6,58^ However, despite the progress made to date, there are few examples in which structure–function relationships have been demonstrated in engineered biomolecular catalysts,^20,23,45,59,60^ especially for systems that exhibit high activity and robustness (*i.e.*, high TON values).

Prior investigation of the mechanism of electrochemical proton reduction by CoMC6*a revealed that proton delivery to CoMC6*a is slow relative to CoMP11-Ac and is impacted by steric hindrance of the proton donor.^48^ The data are consistent with the requirement of a conformational rearrangement of CoMC6*a to facilitate proton delivery, *i.e.*, to expose the distal side of the porphyrin, which is protected by a helix in the folded mini-protein (Fig. 1). In contrast, CoMP11-Ac reacts with proton donors in a diffusion-controlled manner, provided the proton donor has a p*K*_a_ below ∼7.5.^49^ Those results revealed the impact of the distal helix on H_2_ evolution reactivity of CoMC6*a: it slows proton delivery, changes mechanism, and increases catalyst robustness, as reflected by TON_H_2__ values nearly 10-fold higher (230 000) than what is seen for CoMP11-Ac (25 000).^45,61^

Given the more hydrophobic nature of the CoMC6*a active site relative to CoMP11-Ac, we hypothesized that it may display greater CO_2_ reduction activity and/or selectivity compared to CoMP11-Ac. This prediction is consistent with reports that hydrophobic microenvironments can improve activity and selectivity for CO_2_ reduction in MOF- and materials-based catalytic systems.^14,62–64^ and also for catalysts within protein environments.^20,23^

For electrocatalytic CO_2_ reduction at −1.2 V, CoMP11-Ac^22^ and CoMC6*a (Table 1) yield similar and high selectivities for CO production (Table S2† compares results on these catalysts). For CoMP11-Ac at −1.2 V in the presence of MOPS, CHES, or CAPS buffers, values of FE_CO_ range from 81 to 88%, and FE_H_2__ ranges from 5 to 8%, similar to the respective ranges for CoMC6*a (73–86% and 4–11%). The measure that does change when comparing these catalysts under these conditions is TON measured in 2-hour experiments; CoMP11-Ac generally has higher TON values for both H_2_ and CO production at −1.2 V, by a factor of four- to six-fold for CO production and two- to seven-fold for H_2_ production, suggesting that the more solvent-accessible active site of CoMP11-Ac facilitates reaction turnover at −1.2 V. However, when CPE is run at −1.2 V for 24 hours (Fig. S12†), the gap in TON values for CO production between these catalysts closes, with a TON_CO_ of 14 000 for CoMC6*a compared to 32 000 for CoMP11-Ac (Table S6†). This result is attributed to a loss of overall activity for CoMP11-Ac in this longer experiment, in which it yields FE_CO_ of 61% compared to 86% for CoMC6*a. We propose that the more protected nature of the CoMC6*a active site maintains catalyst integrity and activity in this longer experiment. Its total value of FE_H_2__ + FE_CO_ is 91%, but this value is only 70% for CoMP11-Ac. We propose that catalyst degradation, which is significant for CoMP11-Ac, accounts for the balance of FE, consistent with the observation that CoMP11-Ac undergoes deactivation and degradation in longer CPE experiments.^61^ These results illustrate how supermolecular structure confers advantages for CoMC6*a catalysis that translate to it maintaining high activity and selectivity for CO production in longer (24-hour) experiments.

These differences in selectivity between these catalysts change substantially for reactions run at more negative potential. At −1.4 V in the three different buffers, CoMC6*a has FE_CO_ values that vary little (67–76%), while FE_CO_ is lower and more variable (21–48%) for CoMP11-Ac. FE_H_2__ values differ significantly between these two catalysts at −1.4 V, ranging from 4 to 24% for CoMC6*a and 29–63% for CoMP11-Ac in the three buffers. Overall, for both catalysts, a decreased buffer acid p*K*_a_ is correlated with a higher FE_H_2__. We also see that the TON_CO_ value for CoMC6*a at −1.4 V is highest with the least acidic proton donor (CAPS), but for CoMP11-Ac, TON_CO_ at −1.4 V with CAPS is its lowest value among the three buffers. While the basis for this difference is speculative, we propose that these observations support the proposal that the protected and hydrophobic active site of CoMC6*a facilitates CO_2_ binding and inhibits proton delivery to both enhance CO production and inhibit H_2_ evolution, especially at lower potentials that enhance H_2_ evolution activity. However, in CoMP11-Ac, with its solvent-exposed distal site, the p*K*_a_ of the proton donor is the key factor determining overall catalytic activity, such that CO production activity (TON) increases with a more acidic proton donor even as FE_CO_ decreases.

## Conclusions

CoMC6*a is a synthetic mini-enzyme that electrochemically catalyzes CO_2_ reduction to CO in water. We provide evidence that its selectivity for CO_2_ over proton reduction is enhanced relative to CoMP11-Ac, particularly at more negative potentials, which we attribute to protection of its active site and its lower Co(ii/i) potential. The catalytic mechanism for CO formation requires CO_2_ binding before or coupled with Co(ii) reduction for CO formation. CoMC6*a displays an outstanding TON_CO_ of 14 000 over 24 hours and excellent selectivity of 86 : 5 CO : H_2_ products in the same 24-hour experiment, demonstrating that a small artificial biocatalyst can be active, robust, and selective for CO_2_ reduction in water. Furthermore, the activity of CoMC6*a is minimally impacted by air, an unusual and desirable property for a CO_2_ reduction catalyst.

## Data availability

Data supporting this article have been published as ESI.†

## Author contributions

Conceptualization: JLA-H, KLB, AL; funding acquisition: AAS, KLB, AL; investigation: AAS, JLA-H, LL, KBR; supervision: KLB, AL; writing – original draft: AAS, JLA-H; writing – review & editing: KLB, AL, LL.

## Conflicts of interest

There are no conflicts to declare.

## Supplementary Material

SC-OLF-D4SC07026G-s001
