# Supplementary material for: Electrocatalytic CO2 reduction by a cobalt porphyrin mini-enzyme
Source: Chem Sci. 2025 Feb 25;16(13):5707–16. doi: 10.1039/d4sc07026g (PMC11877352; doi:10.1039/d4sc07026g)
Supplement: SC-016-D4SC07026G-s001 [file SC-016-D4SC07026G-s001.pdf]

Supplementary Information (SI) for Chemical Science.
This journal is © The Royal Society of Chemistry 2025

Supporting Information

Electrocatalytic CO_2 reduction by a cobalt porphyrin mini-enzyme

Alison A. Salamatian[a], Jose L. Alvarez-Hernandez[a], Karishma B. Ramesh[a], Linda Leone[b], Angela Lombardi[b], Kara L. Bren*[a]

[a]*Department of Chemistry, University of Rochester, Rochester, NY 14627-0216, USA*

[b]*Department of Chemical Sciences, University of Naples Federico II, Complesso Universitario Monte S. Angelo, via Cintia 45, 80126 Naples, Italy*

*email: kara.bren@rochester.edu

<u>Table of Contents</u>

Experimental details………………………………………………………………………. S2
Comparison data on related systems ……..…………………………………..………….. S8
Results of CPEs of CoMC6*a in MOPS, CHES, CAPS………………………………….. S10
Additional CV data ……………………………………………………………………….. S17
Extended (24-hour) CPE experiments …………………………………………………. S19
GC calibration curves and chromatogram ……………………………………………... S20

Experimental details

CoMC6*a preparation and characterization

CoMC6*a was prepared and purified as previously described.[1, 2] CoMC6*a stock solutions in doubly deionized water were stored at -80 ˚C. The concentration of CoMC6*a was determined from UV-vis spectroscopy in doubly deionized water using the extinction coefficient previously reported.[1, 3]

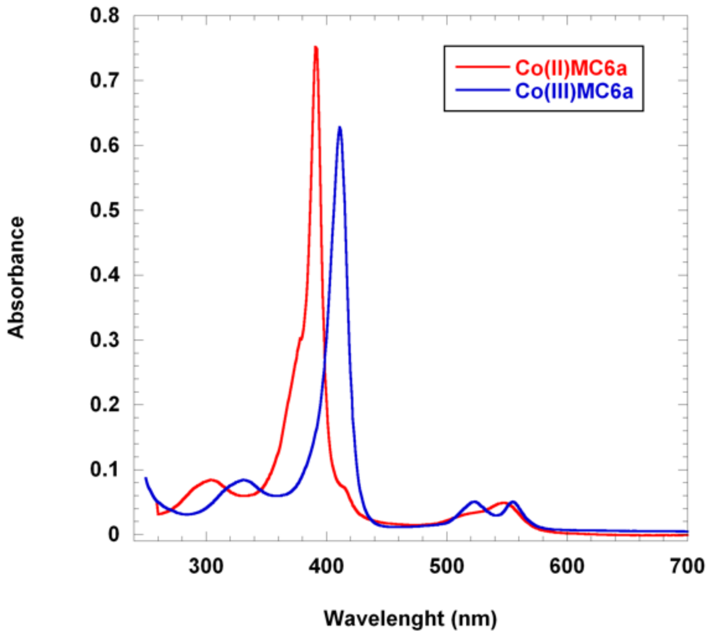

Figure S1. UV-vis absorption spectra of 4.2 μM Co(III)MC6*a (blue) and Co(II)MC6*a (red) in H_2O, 0.1% TFA (pH 2). Co(II)MC6*a was obtained by treating the solution with excess sodium dithionite.

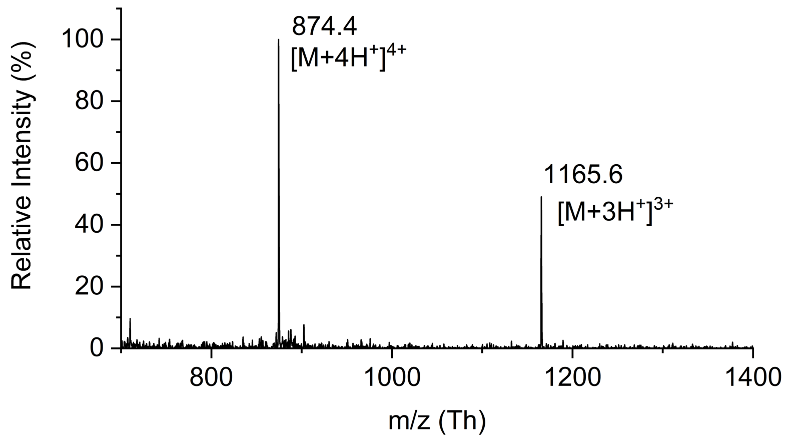

Figure S2. ESI-MS spectrum of Co^{III}-MC6*a. The peaks at m/z 1165.6 and 874.4 correspond to the $[M+3H^{+}]^{3+}$ and $[M+4H^{+}]^{4+}$ ions, respectively. These values are consistent with theoretical mass value of 3493 Da.

Cyclic voltammetry

All cyclic voltammetry (CV) experiments were conducted on a CH Instruments 620D or 720E potentiostat with automatic iR compensation enabled. Experiments used a three-electrode configuration with a Ag/AgCl/$KCl_{(1M)}$ reference electrode (CHI111), a Pt wire counter electrode (CHI115, surface area in solution ~0.14 cm^2), and a mercury drop electrode (BASi CGME MF-9058 used in static mode) as the working electrode (surface area ~2.45×10^{-2} cm^2). Voltammograms were collected by first by scanning from higher potential to lower (more negative) potential, and then back to the less negative potential. The use of a mercury electrode negates concerns about nanoparticle formation because mercury amalgamates cobalt. The CV working solution was purged with N_2 for at least 5 minutes and, for experiments under CO_2, an additional 15 minutes with CO_2 before each experiment. The cell was kept under an atmosphere of N_2 or CO_2 during experiments. CV scans were collected from 0 V to negative potentials and then back to 0 V. For experiments under CO_2, pH of solutions containing 4-morpholinepropanesulfonic acid (MOPS) was initially adjusted to ~ 7. The pH of solutions of 3-(cyclohexylamino)-1-ethanesulfonic acid (CHES) was initially adjusted to ~ 9. The pH of solutions

of 3-(cyclohexylamino)-1-propanesulfonic acid (CAPS) was initially adjusted to ~ 10.5 to enhance solubility. Purging the solutions used in experiments CO_2 then changed the pH of the solution to ~6 from these starting points. The pH values of samples under N_2 was adjusted to match the pH values of the corresponding experiments under CO_2 using small (microliter) amounts of concentrated HCl.

Dip-and-stir test

The dip-and-stir test is a version of the typical rinse test, adapted to a mercury drop electrode.{Alvarez Bren 2021} After a single CV scan is collected on a mercury drop exposed to the catalyst containing solution, or after exposing the mercury drop to the catalyst solution for one minute, the electrochemical cell is removed and the counter and reference electrodes are carefully wiped, while the mercury drop remains at the tip of the capillary. The electrodes are then dipped into an electrochemical cell containing fresh solution with no catalyst. The solution is stirred for 3 minutes using a magnetic stir bar to remove any catalyst-containing droplets from the electrodes and purged with either CO_2 for 5 minutes. A CV is then collected and any above-background activity detected is due to catalyst adsorbed to the mercury drop. More information regarding this adsorption test can be found in reference {Alvarez Bren 2021}.

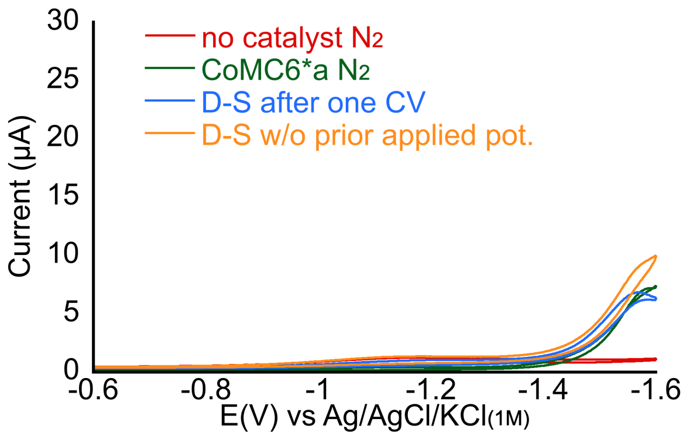

Figure S3. Results of dip-and-stir test for 1 μM CoMC6*a in 50 mM CAPS pH 6.5 under 1 atm N_2. Scan rate 100 mV/s

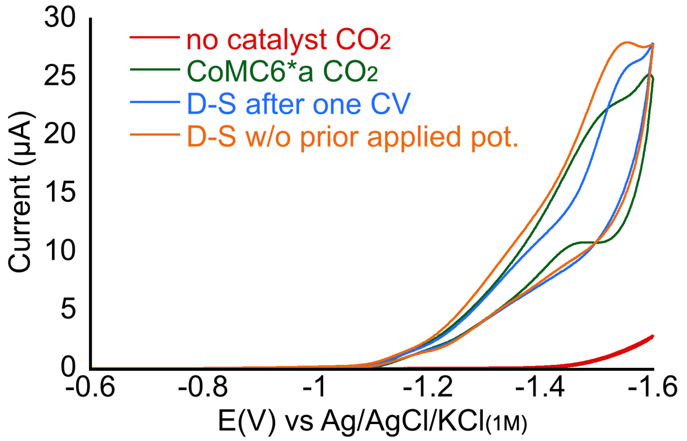

Figure S4. Results of dip-and-stir test for 1 μM CoMC6*a in 50 mM CAPS pH 6.5 under 1 atm CO_2. Scan rate 100 mV/s

Controlled potential electrolysis

Controlled potential electrolysis (CPE) were performed on a CH Instruments 620D or 720E potentiostat. Experiments were performed in a two-compartment cell (H-cell) with a P5 glass frit

with average pore size of 1.0 – 1.5 μm. The working compartment contained the reference Ag/AgCl/KCl$_{(1M)}$ electrode, and working mercury pool electrode with a surface area of ~ 2.0 cm^2, connected to the circuit by an insulated platinum wire. The counter compartment contained a glassy carbon rod counter electrode (surface area ~ 2.5 cm^2). Buffers (500 mM MOPS, CHES, or CAPS, with 1 M KCl) were prepared by dissolving solid in doubly deionized water (prepared with a NanoPure system) and adjusted to the desired pH with HCl or NaOH. The working and counter compartments were filled with 5 mL and 6 mL of solution, respectively, to allow the appropriate surface area of contact with the counter electrode. The solutions were sparged with N_2 (5 minutes) then with 95:5% CO_2/CH_4 (15 minutes; Airgas) or with 80:20% N_2/CH_4 (20 minutes; Airgas) prior to each experiment. The CH_4 served as an internal standard. CPE experiments in the presence of MOPS were conducted as previously reported, with the H-cell headspace connected with 2 needles and fine tubing.[4] Solutions of MOPS, CHES, and CAPS were initially adjusted to pH 7, 9, 10.5, respectively; after purging with CO_2, pH stabilized at 6.5 ± 0.2. For experiments under N_2, all solutions were initially prepared at pH 7, and then adjusted to match the pH of the corresponding experiment conducted under CO_2. CPE data was background corrected, where the background consisted of the CPE experiment run under the same conditions but without catalyst present.

Preparation of samples for investigation of air tolerance

To prepare solutions with dissolved CO_2 but under different headspace gases, a solution was first sparged with CO_2 for 15 minutes, after which before the headspace was purged with either N_2 or air for 5 minutes.

Gas chromatography

A gas chromatography-2014 Fuel Cell Analyzer (Shimadzu) with a methanizer and thermal conductivity and flame ionization detectors was used to determine the amount of H_2 and CO present in headspace gas. Known volumes of H_2 and CO were injected alongside CH_4 as an internal standard to produce calibration curves. A unique calibration curve was produced for each type of reaction vessel purged with the desired gas (N_2 or CO_2 with methane) and containing doubly deionized water at the respective volume of the reaction solution. Determination of turnover number (TON) and faradaic efficiency (FE) values was as previously described.[4]

Formate analysis

Analysis for formate formation used the Formate Assay Kit from Sigma-Aldrich according to the manufacturer's instructions.

Comparison data on related systems

Table S1. Comparison of the activity and selectivity of engineered biomolecular catalysts for CO_2 reduction.

CO_2 Reduction Semi-synthetic Biomolecular Catalysis in Water						
**Catalyst**	**Photosensitizer**	**TON_{CO}**	**TON_{H2}**	**Selectivity**	**Runtime (h)**	**Reference**
Ni(cyclam)@Cu-azurin	$[Ru(bpy)_3]^{2+}$	38<br>12	NA<br>140	NA<br>8% (78% for Zn-azurin)	5<br>2	Chem. Commun. 2016, 52, 9889-9892.
PSP-Ni(II)–terpy (PSP2T2)		120	0	100%	24	Nat. Chem. 2018, 10, 1201–1206
Co-Cyt b_{562}	$[Ru(bpy)_3]^{2+}$	42	143	23%	8	Frontiers in Molecular Biosciences 2021, 8 (17)
(A3A3′)Y26C-Co(III)Mal-PPIXMME	$[Ru(bpy)_3]^{2+}$	80*	163*	33%*	~20	Int. J. Mol. Sci. 2022, *23*, 14640.
CoMb	$[Ru(bpy)_3]^{2+}$	2000 (280)	11300 (100)	15% (74%)	2	Angew. Chem. 2023, e202215719
CoMP11-Ac	Electrochemical	32,000 (1500)	4,600 (80)	61% (88%)	24 (2)	ACS Catal. 2022, 12, 23, 14689–14697

*Results were not provided for the same experimental conditions for H_2 and CO

Table S2. Comparison of results on CoMC6*a and CoMP11-Ac[a]

	Buffer	Catalyst	$FE_{(H_2)}$ %	$FE_{(CO)}$ %	$TON_{(H_2)}$	$TON_{(CO)}$	Q_T (C)
**CO_2 -1.4 V** [b]	CAPS (pK_a 10.4)	CoMP11-Ac	29 ± 6	48 ± 10	280 ± 10	470 ± 10	0.9 ± 0.2
		CoMC6*a	4 ± 1	76 ± 10	110 ± 20	2100 ± 600	2.6 ± 0.4
	CHES (pK_a 9.3)	CoMP11-Ac	43 ± 9	57 ± 4	940 ± 30	1300 ± 300	2.2 ± 0.3
		CoMC6*a	14 ± 1	67 ± 12	280 ± 10	1300 ± 400	1.9 ± 0.1
	MOPS (pK_a 7.2)	CoMP11-Ac	63 ± 13	21 ± 5	4100 ± 500	1400 ± 500	6.4 ± 0.8
		CoMC6*a	24 ± 4	68 ± 8	390 ± 120	1100 ± 200	1.6 ± 0.5
**CO_2 -1.2 V** [b]	CAPS (pK_a 10.4)	CoMP11-Ac	5 ± 1	88 ± 11	80 ± 20	1500 ± 300	1.7 ± 0.6
		CoMC6*a	4 ± 4	73 ± 5	11 ± 10	230 ± 10	0.3 ± 0.1
	CHES (pK_a 9.3)	CoMP11-Ac	6 ± 1	81 ± 2	250 ± 30	3500 ± 300	4.2 ± 0.4
		CoMC6*a	11 ± 1	86 ± 11	100 ± 20	800 ± 200	0.9 ± 0.1
	MOPS (pK_a 7.2)	CoMP11-Ac	8 ± 2	85 ± 2	1200 ± 100	12000 ± 1000	14.1 ± 1.4
		CoMC6*a	6 ± 1	85 ± 11	160 ± 40	2200 ± 300	2.5 ± 0.2

[a]Results on CoMP11-Ac from SI ref. 4. Two-hour CPE experiments were conducted on 1 μM catalyst in 0.5 M buffer with 1 M KCl. Data shown corresponds to the average of at least three individual runs, the error corresponds to the difference between the average and the replicate with the greatest difference from the average. Activity is not reported if it did not exceed three times background in more than one replicate. The pH of all solutions under CO_2 6.5
[b]Potentials reported vs. Ag/AgCl/KCl(1M).

Results of CPEs of CoMC6*a in MOPS, CHES, CAPS

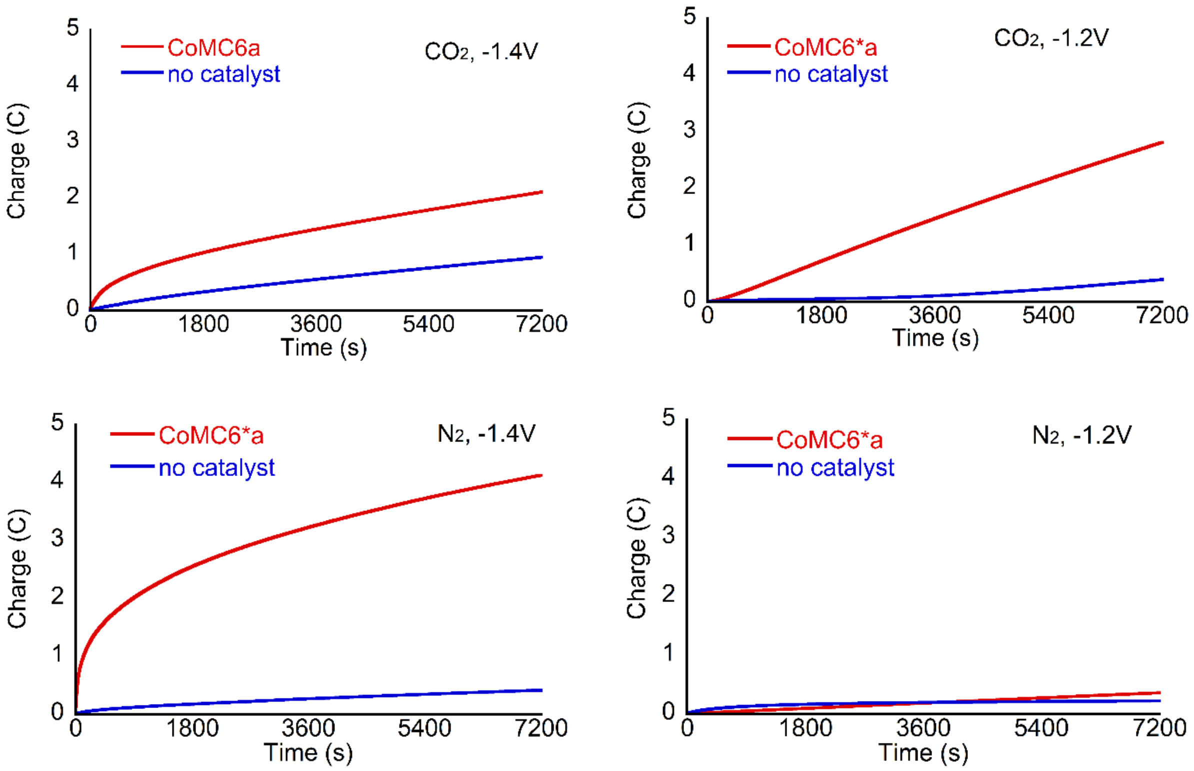

Figure S5. CPE experiments run in 0.5 M MOPS, 1 M KCl, and 1 μM CoMC6*a, when present. Results, as labeled in the figure, are from CPE run in CO_2-saturated solution at −1.4 V (upper left), CO_2-saturated solution at −1.2 V (upper right), N_2-saturated solution at −1.4 V (lower left), and N_2-saturated solution at −1.2 V (lower right). The pH of all MOPS and CAPS solutions after purging with CO_2 was 6.5 ± 0.1; and 7.2 ± 0.2 when purged with N_2. Potentials are reported vs. Ag/AgCl/KCl$_{(1M)}$.

Table S3. Results of 2-hour CPE experiments on 1 μM CoMC6*a in 0.5 M MOPS, 1 M KCl[a]

	E (V)	FE(H_2)	FE(CO)	TON(H_2)	TON(CO)	Q_T (C)
CO_2	-1.4	26	69	500	1300	1.8
		24	60	510	1300	2.1
		24	77	310	980	1.2
		20	70	270	960	1.3
		25	66	340	910	1.3
	-1.2	7	94	190	2500	2.5
		5	74	130	1900	2.5
		7	86	190	2400	2.7
		5	87	120	2200	2.4
N_2	-1.4	99	1	5400	49	5.3
		97	1	2900	34	2.8
		92	1	3500	52	3.6
	-1.2	No above-background activity[b]				

[a]Two-hour CPE experiments conducted for 1 μM catalyst in 0.5 M buffer with 1 M KCl. The pH of all MOPS and CAPS solutions after purging with CO_2 was 6.5 ± 0.1; and 7.2 ± 0.2 when purged with N_2. Potentials reported vs. Ag/AgCl/KCl$_{(1M)}$. [b]Activity is not reported if activity was not three times background in more than one replicate.

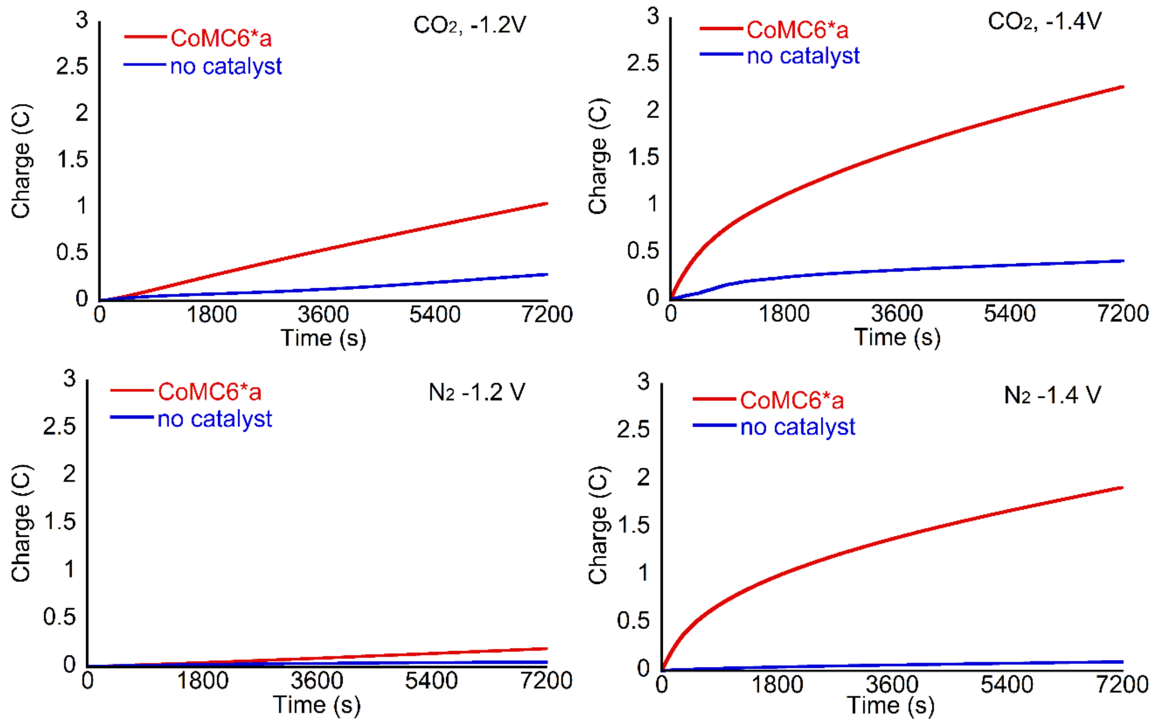

Figure S6. CPE experiments run in 0.5 M CHES, 1 M KCl, the concentration of catalyst was 1 µM when present. CPE run in (a) CO_2-saturated solution at -1.4 V, (b) CO_2-saturated solution at -1.2 V, (c) N_2-saturated solution at -1.4 V, and (d) N_2-saturated solution at -1.2 V. pH = 6.6 ± 0.1 for (a) and (b), and 7.1 ± 0.3 for (c) and (d). Potentials reported vs. Ag/AgCl/$KCl_{(1M)}$

Table S4. Results of 2-hour CPEs of 1 μM CoMC6*a in 0.5 M CHES, 1 M KCl[a]

	E (V)	$FE(H_2)$	FE(CO)	$TON(H_2)$	TON(CO)	Q_T (C)
CO_2	-1.4	14	60	270	1100	1.8
		13	79	280	1700	2.0
		15	62	290	1200	1.9
	-1.2	11	81	88	646	0.8
		11	97	120	1000	1.0
		10	79	87	710	0.9
N_2	-1.4	~100	~0	1900	~0	1.8
		83	~0	1600	~0	1.8
		~100	~0	1900	~0	1.8
	-1.2	66	~0	100	~0	0.1
		92	~0	130	~0	0.1
		76	~0	160	~0	0.2

.

[b]Two-hour CPE experiments conducted for 1 μM catalyst in 0.5 M buffer with 1 M KCl. The pH of all MOPS, CHES and CAPS solutions after purging with CO_2 was 6.5 ± 0.1; and 7.2 ± 0.2 when purged with N_2. Potentials reported vs. Ag/AgCl/$KCl_{(1M)}$.

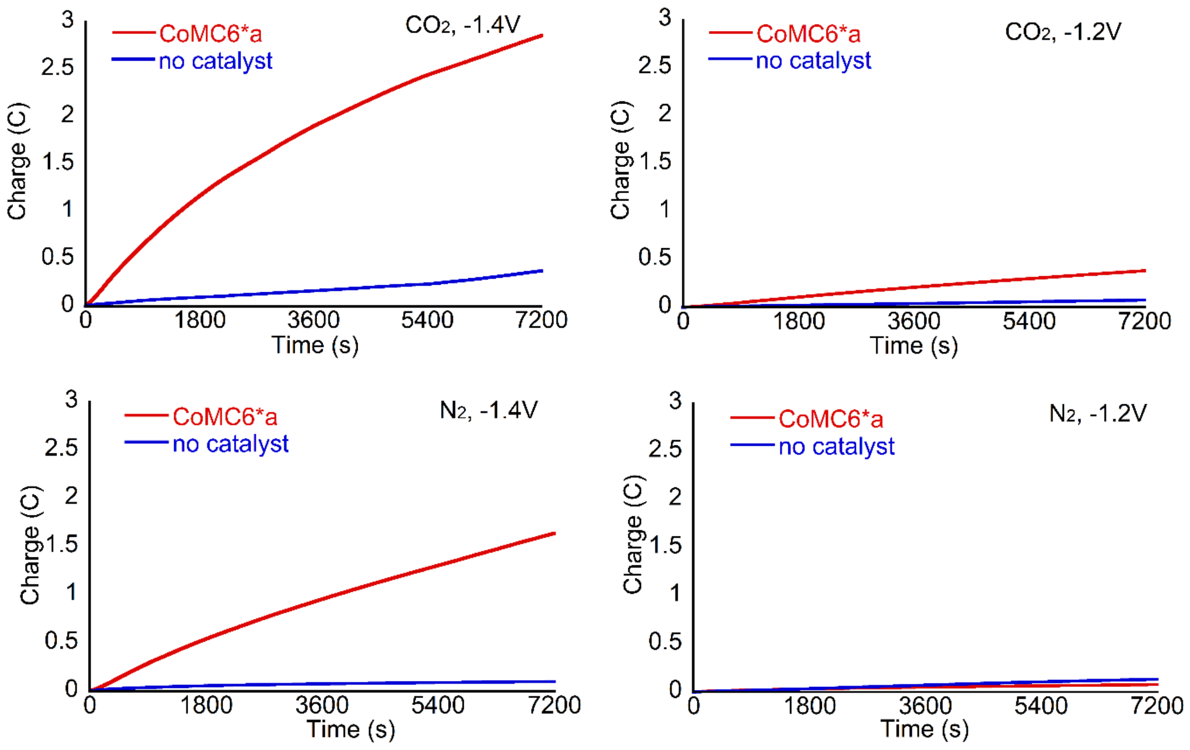

Figure S7. CPE experiments run in 0.5 M CAPS, 1 M KCl, the concentration of catalyst was 1 μM when present. CPE run in (upper left) CO_2-saturated solution at -1.4 V, (upper right) CO_2-saturated solution at -1.2 V, (lower left) N_2-saturated solution at -1.4 V, and (lower right) N_2-saturated solution at -1.2 V. The pH of all MOPS and CAPS solutions after purging with CO_2 was 6.5 ± 0.1; and 7.2 ± 0.2 when purged with N_2. Potentials reported vs. Ag/AgCl/KCl$_{(1M)}$.

Table S5. Results of 2-hour CPE experiments on 1 μM CoMC6*a in 0.5 M CAPS, 1 M KCl[a]

	E (V)	$FE(H_2)$	FE(CO)	$TON(H_2)$	TON(CO)	Q_T (C)
CO_2	-1.4	4	85	130	2600	2.9
		4	77	120	2100	2.6
		4	73	120	2000	2.7
		4	69	97	1500	2.2
	-1.2	6	77	18	230	0.4
		5	73	16	220	0.4
		0	68	0	240	0.4
N_2	-1.4	91	~0	810	~0	1.0
		96	~0	1500	~0	1.6
		76	~0	970	~0	1.4
	-1.2	No above-background activity[b]				

[a]Two-hour CPE experiments conducted for 1 μM catalyst in 0.5 M buffer with 1 M KCl. The pH of all MOPS and CAPS solutions after purging with CO_2 was 6.5 ± 0.1; and 7.2 ± 0.2 when purged with N_2. Potentials reported vs. Ag/AgCl/$KCl_{(1M)}$. [b]Activity is not reported if activity was not three times background in more than one replicate.

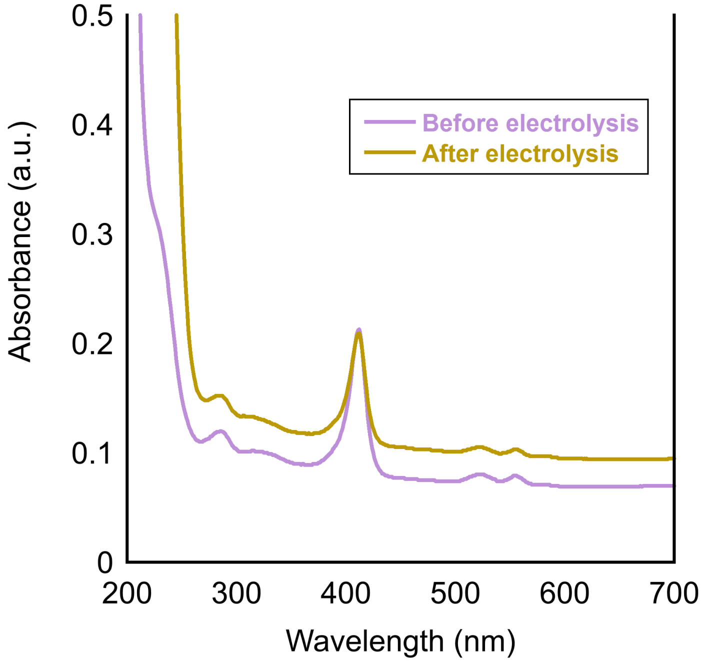

Fig. S8. UV-Vis spectra of 1 μM CoMC6*a taken in 1 M KCl before and after bulk electrolysis at -1.2 V for 2 hrs, with CO_2/CH_4 purged for 15 minutes. The data were collected in the absence of MOPS, CHES, or CAPS buffer because oxidation of the buffers during catalysis yields a species that interferes with the absorption measurement.

Additional CV data

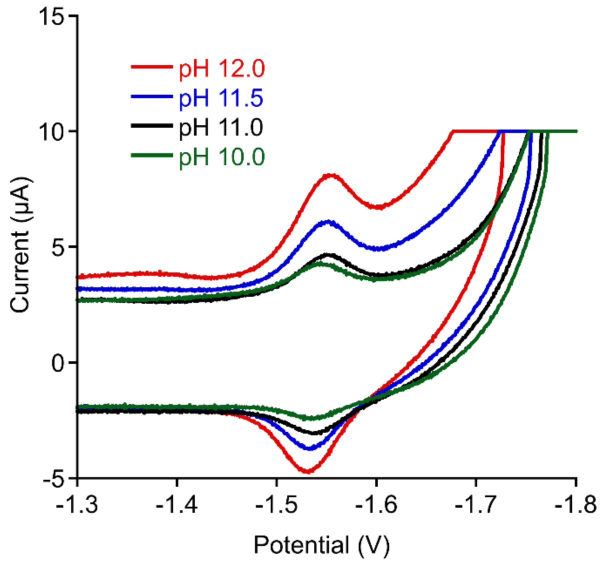

Fig S9. CVs of 1 µM CoMC6*a in 0.1 M KCl, under 1 atm N_2 at 5 V/s at pH 10-12 as indicated.

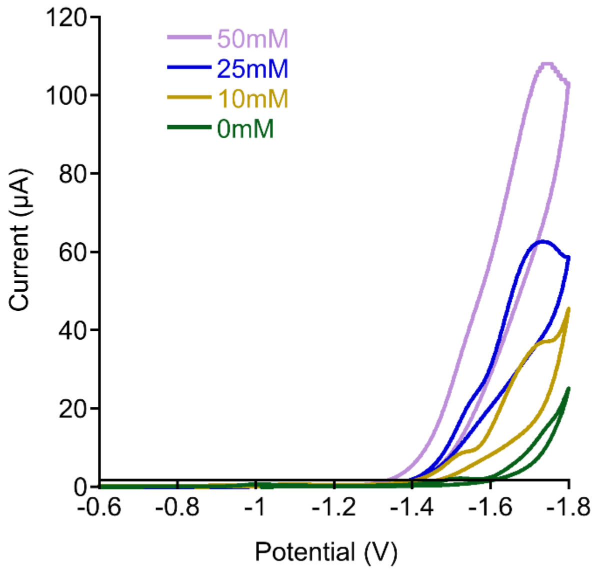

Fig S10. CVs of 1 μM CoMC6*a in different CHES concentrations with 0.1 M KCl, pH 6.3 ± 0.05, under 1 atm N_2, at 100mV/s.

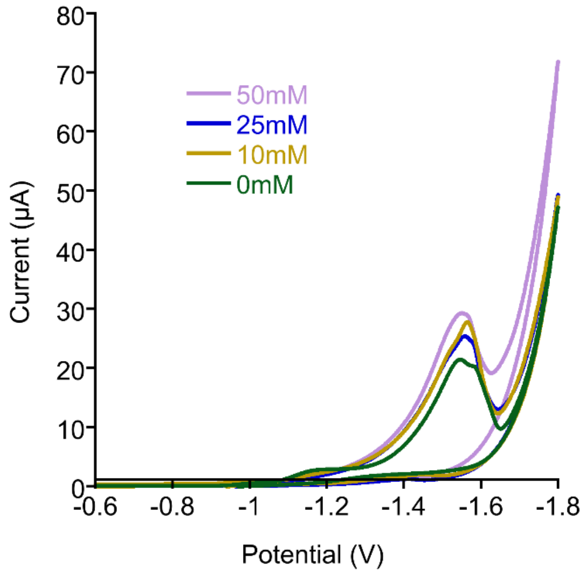

Fig S11. CVs of 1 μM CoMC6*a in different CHES concentrations with 0.1 M KCl, pH 6.3 ± 0.05, under 1 atm CO_2, at 100mV/s.

Extended (24-hour) CPE experiments

Table S6. Results of 24-hour CPE experiments on1 μM indicated catalyst in 0.5 M MOPS, 1 M KCl under CO_2[a]

Buffer	E (V)[b]	FE(H_2)	FE(CO)	TON(H_2)	TON(CO)	Q_T (C)
CoMC6*a	-1.2	5 ± 1	86 ± 5	810 ± 170	14,000 ± 60	16 ± 1
CoMP11-Ac[4]	-1.2	9 ± 2	61 ± 15	4600 ± 300	32,000 ± 9000	51 ± 7

[a]Data shown corresponds to the average of at least two individual runs. [b]Potentials reported vs. Ag/AgCl/KCl$_{(1M)}$.

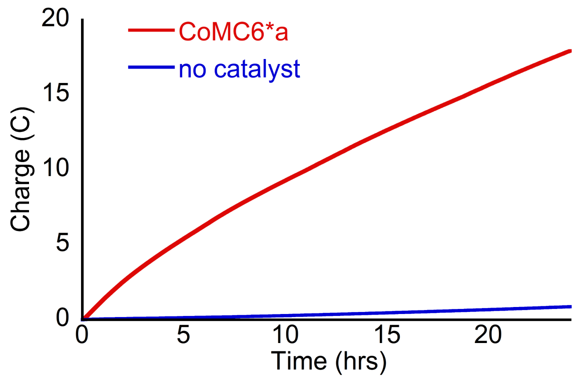

Figure S12. CPE experiments run in 0.5 M MOPS, 1 M KCl, under CO_2 at -1.2 V, the concentration of CoMC6*a was 1 μM when present.

GC calibration curves and chromatogram

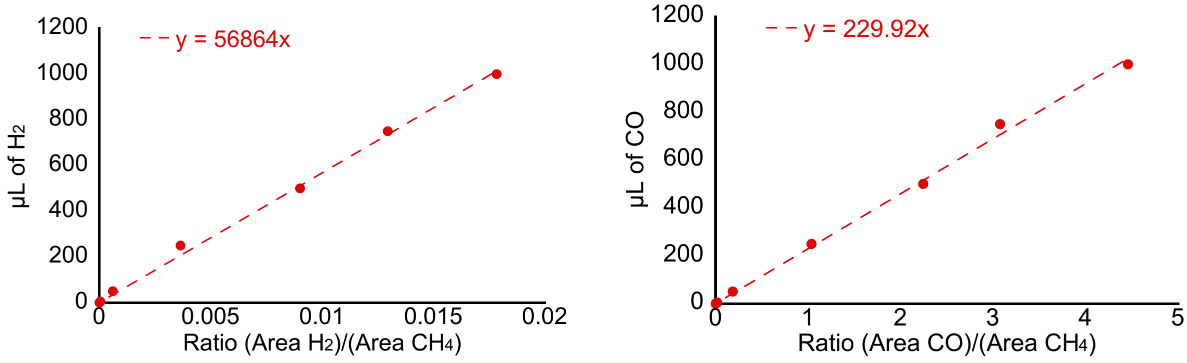

Figure S12. Calibration curves for the quantification of H_2 (left) and CO (right), with 95:5% CO_2:CH_4 as the purging gas.

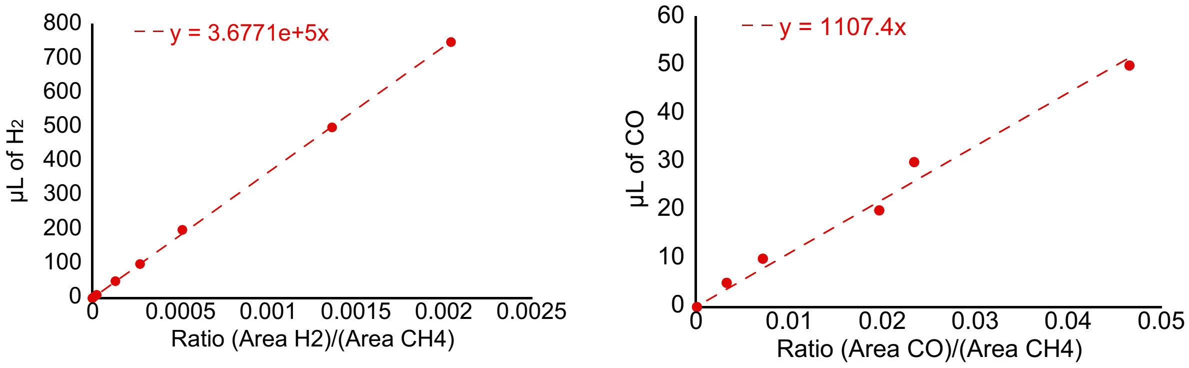

Figure S13. Calibration curves for the quantification of H_2 with 80:20% N_2:CH_4 as the purging gas. (Left) H_2 (Right) CO.

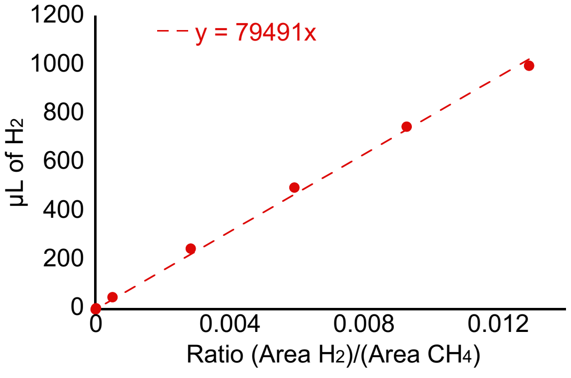

Figure S14. Calibration curve for the quantification of H_2 with 95:5% CO_2:CH_4 as the purging gas in an H-cell with connected headspaces.

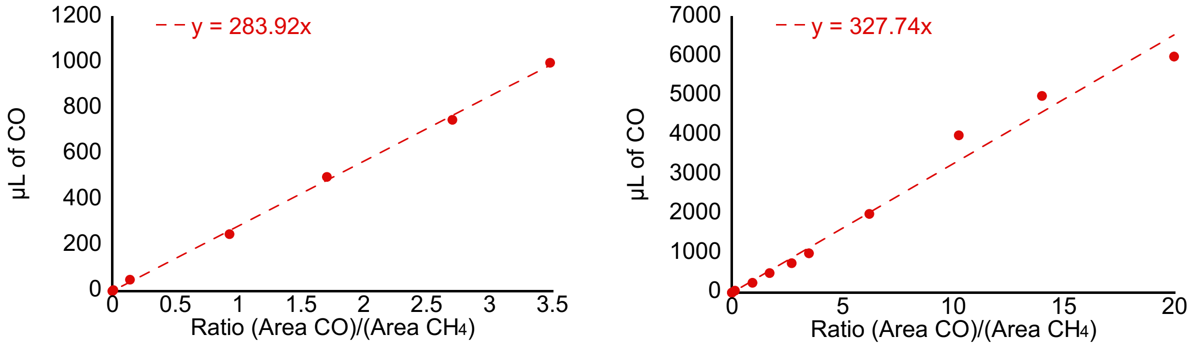

Figure S15. Calibration curves for the quantification of CO with 95:5% CO_2:CH_4 as the purging gas in an H-cell with connected headspaces. (Left) Low volumes. (Right) High volumes.

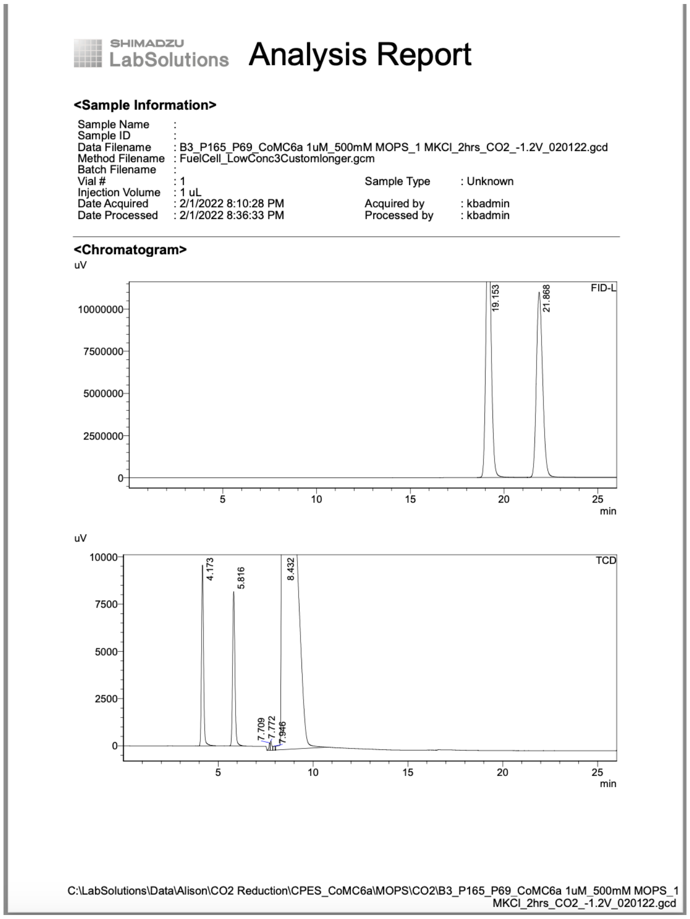
SHIMADZU LabSolutions

Analysis Report

<Sample Information>

Sample Name :
Sample ID :
Data Filename : B3_P165_P69_CoMC6a 1uM_500mM MOPS_1 MKCl_2hrs_CO2_-1.2V_020122.gcd
Method Filename : FuelCell_LowConc3Customlonger.gcm
Batch Filename :
Vial # : 1 Sample Type : Unknown
Injection Volume : 1 uL
Date Acquired : 2/1/2022 8:10:28 PM Acquired by : kbadmin
Date Processed : 2/1/2022 8:36:33 PM Processed by : kbadmin

<Chromatogram>

C:\LabSolutions\Data\Alison\CO2 Reduction\CPES_CoMC6a\MOPS\CO2\B3_P165_P69_CoMC6a 1uM_500mM MOPS_1 MKCl_2hrs_CO2_-1.2V_020122.gcd

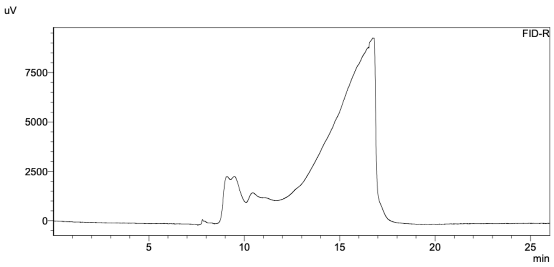

<Peak Table>

FID-L

Peak#	Ret. Time	Area	Height	Conc.	Unit	Mark	Name
1	19.153	304655425	16210406	48158.749	ppm		methane
2	21.868	254073071	10992799	34509.876	ppm	SV	CO
Total		558728496	27203204				

TCD

Peak#	Ret. Time	Area	Height	Conc.	Unit	Mark	Name
1	4.173	71731	9567	3112.673	ppm		H2
2	5.816	69230	8162	30474.106	ppm		O2
3	7.709	1643	400	0.000			
4	7.772	2804	496	0.000		V	
5	7.946	1689	207	0.000		V	
6	8.432	1140370	26704	0.000	ppm	V	N2
Total		1287467	45535				

FID-R

Peak#	Ret. Time	Area	Height	Conc.	Unit	Mark	Name
Total							

C:\LabSolutions\Data\Alison\CO2 Reduction\CPES_CoMC6a\MOPS\CO2\B3_P165_P69_CoMC6a 1uM_500mM MOPS_1 MKCl_2hrs_CO2_-1.2V_020122.gcd

Figure S16. Example of GC chromatogram

References

1. V. Firpo, J. M. Le, V. Pavone, A. Lombardi and K. L. Bren, Hydrogen evolution from water catalyzed by cobalt-mimochrome VI*a, a synthetic mini-protein, *Chem. Sci.*, 2018, **9**, 8582-8589.
2. G. Caserta, M. Chino, V. Firpo, G. Zambrano, L. Leone, D. D'Alonzo, F. Nastri, O. Maglio, V. Pavone and A. Lombardi, Enhancement of Peroxidase Activity in Artificial Mimochrome VI Catalysts through Rational Design, *ChemBioChem*, 2018, **19**, 1823-1826.
3. E. H. Edwards, J. M. Le, A. A. Salamatian, N. L. Peluso, L. Leone, A. Lombardi and K. L. Bren, A cobalt mimochrome for photochemical hydrogen evolution from neutral water, *J. Inorg. Biochem.*, 2022, **230**, 111753.
4. J. L. Alvarez-Hernandez, A. A. Salamatian, J. W. Han and K. L. Bren, Potential- and Buffer-Dependent Selectivity for the Conversion of CO_2 to CO by a Cobalt Porphyrin-Peptide Electrocatalyst in Water, *ACS Catal.*, 2022, DOI: 10.1021/acscatal.2c03297, 14689-14697.
